# Effectiveness of Trauma-Focused Psychological Therapies for Treating Post-traumatic Stress Disorder Symptoms in Women Following Childbirth: A Systematic Review and Meta-Analysis

**DOI:** 10.3389/fpsyt.2018.00591

**Published:** 2018-11-20

**Authors:** Marie Furuta, Antje Horsch, Edmond S. W. Ng, Debra Bick, Debbie Spain, Jacqueline Sin

**Affiliations:** ^1^Department of Human Health Sciences, Graduate School of Medicine, Kyoto University, Kyoto, Japan; ^2^Institute of Higher Education in Healthcare Research, University of Lausanne and Lausanne University Hospital, Lausanne, Switzerland; ^3^Department Woman-Mother-Child, Lausanne University Hospital, Lausanne, Switzerland; ^4^Director's Office, London School of Hygiene & Tropical Medicine, London, United Kingdom; ^5^Department of Women and Children's Health, Faculty of Life Sciences and Medicine, School of Life Course Sciences, King's College London, London, United Kingdom; ^6^Institute of Psychiatry, Psychology and Neuroscience, King's College London, London, United Kingdom; ^7^School of Psychology & Clinical Language Sciences, University of Reading, Reading, United Kingdom; ^8^Berkshire Traumatic Stress Service, Berkshire Healthcare NHS Foundation Trust, Bracknell, United Kingdom

**Keywords:** perinatal, postpartum, posttraumatic stress, posttraumatic stress disorder (PTSD), trauma-focused, cognitive therapy, psychotherapy, meta-analysis

## Abstract

**Background:** Approximately 3% of women in community samples develop posttraumatic stress disorder (PTSD) after childbirth. Higher prevalence rates are reported for high risk samples. Postpartum PTSD can adversely affect women's wellbeing, mother-infant relationships and child development. This study aims to examine the effectiveness of trauma-focused psychological interventions (TFPT), for postnatal women.

**Methods:** We conducted a systematic review and meta-analysis including all clinical trials which reported post-traumatic stress symptoms for both the intervention and control groups or at least two time-points, pre- and post-intervention. We searched four databases: CENTRAL, MEDLINE, PsycINFO, and OpenGrey. Screening of search results, data extraction, and risk of bias assessment were undertaken independently by two reviewers.

**Results:** Eleven studies, reported in 12 papers, involving 2,677 postnatal women were included. All were RCTs, bar one case series. Interventions varied in modality, duration and intensity, and included exposure therapy, trauma-focused cognitive behavioral therapy, eye movement desensitization and reprocessing and other psychological approaches. Participants had experienced uncomplicated births, emergency cesarean sections and/or preterm births. Results suggest that TFPT are effective for reducing PTSD symptoms in the short term (up to 3 months postpartum [4 RCTs, *n* = 301, SMD = −0.50, 95% CI = −0.73 to −0.27]), and medium term (i.e., 3–6 months postpartum [2 RCTs, *n* = 174, SMD = −1.87, 95% CI = −2.60 to −1.13]). However, there is no robust evidence to suggest whether TFPT can also improve women's recovery from clinically significant PTSD symptoms.

**Conclusion:** Further larger studies, distinguishing between low and high risk groups, and with adequate follow-up, are needed to establish which TFPT are most effective and acceptable for treating postnatal PTSD.

## Introduction

There are ~135 million births worldwide each year (1). For many women, childbirth is a positive and exciting, if not slightly daunting experience. But for some, giving birth can be frightening, anxiety-provoking, and traumatic (2). It is increasingly recognized that a difficult birth experience can result in highly distressing symptoms and posttraumatic stress symptoms that fail to meet the diagnostic threshold, or full-blown posttraumatic stress disorder (PTSD) (3, 4). Symptoms of PTSD include re-experiencing (e.g., intrusive images of witnessing one's own severe blood loss or being rushed to hospital in an emergency situation); avoidance of reminders of the traumatic event (e.g., avoiding hospital appointments if the birth took place at the hospital); negative mood and cognitions (e.g., It's my fault that the birth didn't go according to plan); and hyperarousal (e.g., hypervigilance with regards to her baby) (5). Meta-analyses suggest that 3% of women after childbirth without objective threat to the life of the mother and/or her baby (low risk group) receive a diagnosis of PTSD. However, in cases where an objective threat to the life of the mother and/or her baby exists (high risk group), such as after emergency cesarean section (EmCS) or preterm birth, around 16% of women are diagnosed with PTSD (6).

PTSD following childbirth can negatively affect the experience and outcomes of subsequent pregnancies, with increased risk of maternal stress and its associated risks of intrauterine growth retardation, premature birth, and low birth weight (7, 8). It can lead to a fear of subsequent pregnancy and childbirth (tokophobia), sexual problems, and avoidance of medical care (9, 10). Some women may decide not to have further children (11). Studies show that postpartum PTSD can also have negative consequences for the attachment relationship with the baby (12, 13), and a detrimental impact on infant behavior and cognitive development (14–17). Associations between PTSD symptoms following childbirth and breastfeeding initiation and breastfeeding continuation were recently shown, which may have negative impact on the development of the mother-infant bond and infant development (18). Another prospective study found that maternal postpartum PTSD symptoms 8 weeks after birth were associated with poor social-emotional development at child age 2 years (19). However, a recent systematic review concluded that evidence for an association between maternal PTSD after childbirth and mother-infant interaction, the mother-infant relationship and child development was inconclusive (20). The authors of the systematic review did conclude that maternal postpartum PTSD was associated with low infant birth weight and lower rates of breastfeeding.

It is anticipated that the number of women experiencing traumatic births will rise, due to the increasingly complex medical needs of women who become pregnant, for example, when older and/or obese (21–24). There is, therefore, an impetus to ascertain how best to ensure that relevant healthcare professionals are able to identify potential signs and symptoms of PTSD early in order to offer appropriate treatment for women who suffer from posttraumatic stress symptoms during the postnatal period (25).

### Description of trauma-focused psychological therapies

Trauma-focused psychological therapies (TFPT) are consistently found to be effective for ameliorating PTSD symptoms that result from single-event trauma in the general population (26–28). The Cochrane systematic review on psychological therapies for adult patients with chronic post-traumatic stress (27), for example, reported that individual trauma focused cognitive behavioral therapy (TFCBT) and Eye-Movement Desensitization and Reprocessing (EMDR) were more effective to reduce clinician-rated PTSD symptoms compared to waitlist/usual care (28 studies; *n* = 1,256, standardized mean difference [SMD] = −1.62, 95% confidence intervals [CI] = −2.03 to −1.21; 6 studies; *n* = 183, SMD = −1.17, 95% CI −2.04 to −0.30; respectively) (29). Also reported in their network meta-analysis of 66 trials that trauma-focused psychological interventions (TFPT) for adult patients with PTSD (in particular CBT and exposure therapy) are effective in reducing PTSD symptom severity, though evidence to date did not identify any specific superior intervention. A course of 8–12 individual outpatient sessions of EMDR (30) or trauma-focused CBT, in particular, are recommended by the UK National Institute for Health and Care Excellence (NICE) guidance on PTSD for children and adults who have experienced a single traumatic event (26). Indeed, the NICE clinical guideline on antenatal and postnatal mental health also recommends that TFCBT or EMDR should be offered to women who suffer PTSD resulting from a traumatic birth (31).

Trauma-focused psychological therapies include various forms of exposure therapy, such as narrative exposure therapy (NET), TFCBT, and EMDR (26, 27, 30). TFPT are underpinned by several shared principles, especially, an emphasis on supporting patients to make sense of and process memories of the trauma and identify and reappraise cognitions and attributions relating to traumatic events (32–34). Exposure therapy requires patients to develop a trauma narrative (a detailed account of the event), either in their imagination or by writing this down, as in NET where a patient is supported to process their memory of the traumatic event and write it down in detail over the course of treatment. They are then asked to repeatedly read or revisit this narrative, so as to become habituated to posttraumatic stress symptoms, e.g., autonomic arousal manifesting concurrently (35, 32). In TFCBT, patients are encouraged to make sense of their experiences, to identify thoughts or patterns of thinking that are negative, and to establish which coping responses may be helpful in the short term, but perpetuate symptoms in the longer term. Individuals are supported to develop new ways of thinking about and coping with the trauma (36). In EMDR, patients are supported to identify and focus on a traumatic image (e.g., bleeding heavily and unexpectedly), an associated thought (e.g., “my baby is going to die”), and the accompanying emotion (e.g., extreme fear) and/or physical sensations (e.g., heart palpitations) while receiving bilateral stimulation, most commonly in the form of eye movements (37).

### Rationale

Although TFPT are deemed effective and acceptable as treatments for addressing PTSD symptoms across clinical populations, postpartum women are often not represented in pre-existing studies. This may be due to: the longstanding under-recognition of PTSD in relation to giving birth; the diagnostic overshadowing of postnatal depression and other conditions; and the lack of help-seeking initiated by the women themselves (3, 4). Relatively little is known about the effectivness of TFPT for postpartum women (38). No previous systematic reviews have synthesized empirical data about effectiveness of TFPT for postpartum women. One Cochrane review focusing on psychological and psychosocial interventions for postnatal depression has been published (39, 40), but PTSD and trauma symptoms were not included as outcomes of interest.

It is possible that PTSD following childbirth differs from PTSD occurring in other situations (41). Whereas typical stressors contributing to PTSD are aversive, such as abuse or torture, childbirth is usually a positive event, although for some it is an event which is traumatic. The implication is that women's psychological health needs may be misunderstood (42). Some of the behaviors women may exhibit, such as social withdrawal and avoidance, may be misattributed to needing to care for their infants, when in fact these could be a consequence of PTSD. It is also evident that for some women caring for a baby continues to be a reminder of traumatic experiences, which may in turn jeopardize the propensity for developing strong bonds and secure attachments between mother and child. Overall, it is likely to be clinically important to take account of the postnatal context when planning and delivering TFPT.

### Objectives

This review had three objectives: (1) to synthesize evidence about the effectiveness of TFPT for women following childbirth; (2) to establish whether any intervention approach is more effective; and (3) to outline implications for clinical practice and research. The primary outcome of interest was identified a priori as PTSD symptoms.

## Methods

### Systematic review protocol

We published the review protocol in PROSPERO (International Prospective Register of Systematic Reviews) (CRD42016043897) and in BMJ Open (25). The review process followed PRISMA guidelines (43).

### Search strategy

We devised search terms for the population (e.g., “pregnancy,” “postpartum period”), intervention (e.g., “psychotherapy,” “cognitive therapy”) and study design of the review questions (see Table 1 for the search strategy). Key search terms (i.e., MESH terms) were used in combination with free text to maximize the search sensitivity. We searched for randomized controlled trials (RCT) and other types of clinical trials (including non-comparative studies, pre- and post- studies, case series and/or case studies) reporting (TFPT) received by women following childbirth. We searched for papers published between 1 January 1990 and 27 September 2017 in the following databases: Cochrane Central Register of Controlled Trials (CENTRAL); MEDLINE; PsycINFO; and OpenGrey. Additionally, the reference lists of all included studies and relevant existing systematic reviews were checked for further possible studies.

**Table 1:** Search strategy (Medline).

**Population**	**AND**	**Intervention**	**AND**	**Study design**
exp pregnancy/		exp cognitive therapy/		exp clinical trial/
*OR*		*OR*		*OR*
exp pregnancy outcome/		CBT.mp.		exp controlled clinical trial/
*OR*		*OR*		*OR*
exp delivery, obstetric/		exp eye movement desensitization reprocessing/		exp clinical trial, phase II/
*OR*		*OR*		*OR*
exp pregnancy complications/		EMDR		exp randomized controlled Trial/
*OR*		*OR*		*OR*
exp parturition/		exp behavior therapy/		exp random allocation/
*OR*		*OR*		*OR*
birth.mp		Behavior* therapy.mp		exp psychology, experimental/
*OR*		*OR*		*OR*
childbirth.mp		exp psychotherapy/		exp cohort studies/
*OR*		*OR*		*OR*
exp postnatal care/		psychological.mp		exp case-control studies/
*OR*		*OR*		*OR*
postnatal.mp.		exp psychological techniques/		exp control groups/
*OR*		*OR*		*OR*
exp postpartum period/		exp psychology, experimental/		randomized.mp
*OR*		*OR*		*OR*
postpartum.mp		Trauma focused.mp		trial.mp
*OR*		*OR*		*OR*
exp maternal health services/		exp stress disorders, post-traumatic/		RCT.mp
*OR*				*OR*
exp maternal-child health services/				intervention
*OR*			
exp infant, newborn/			
*OR*			
exp cesarean section/			
*OR*			
cesarean			
*OR*			
exp stillbirth/			
*OR*			
exp intensive care, neonatal/			
*OR*			
exp intensive care units, neonatal/			

### Inclusion and exclusion criteria

The population studied was women after childbirth. There were no restrictions on age, nationality or birth mode. The review included women who had had either an uncomplicated birth (low risk group) or one with complications (high risk group), such as a pre-term birth. We excluded studies which only recruited women who had suffered loss of pregnancy before reaching gestational week 20 (e.g., miscarriage), as this is regarded as a non-childbirth related event.

In terms of interventions, the review focused on trauma-focused treatments or other psychological therapies used with the explicit intention of treating PTSD and related distress resulting from the experience of childbirth. We categorized TFPT into four discrete modalities based on their theoretical underpinnings and treatment approaches, as follows:

1. Exposure therapy: Any therapy targeted at the individual patient which involves guiding them to relive and process their memory of the trauma by creating a written or audio-recorded narrative. During sessions, individuals are asked to repeatedly revisit their narrative to enable habituation (tolerance) of trauma symptoms.
2. Cognitive behavioral therapy which is trauma-focused (TFCBT): Any psychological therapy that predominantly employs trauma-focused cognitive, behavioral or cognitive-behavioral techniques, and supports patients to identify unhelpful thoughts or thinking styles about themselves, others or the world, behaviors, and evolve other ways of coping with trauma.
3. EMDR: This comprises eight elements, including recall of images, thoughts, emotions and bodily sensations associated with traumatic events, while receiving bilateral stimulation. It is a structured protocol-driven trauma-focused therapy, with a basis in an adaptive information process model of PTSD (44).
4. Any other psychological intervention which did not match with the above categories, but described the theoretical underpinning and was planned to target trauma and/or posttraumatic stress symptoms in postpartum women.

#### Comparators (if applicable) reported in the control arms were categorized into two types

1. Usual postnatal care, which refers to the usual postnatal care provided to women within the first 6 weeks post-birth in settings that did not routinely offer TFPT; and
2. Usual postnatal care, plus any *ad-hoc* supportive counseling or “attention control” (e.g., peer support).

We included all clinical trials using any designs as long as outcome measure data on the review primary outcomes (posttraumatic stress symptoms, see below) were reported for at least two time-points, pre- and post-intervention-exposure.

### Outcomes and measures

The primary outcome measures were PTSD or posttraumatic stress symptoms as measured by validated scales such as the Perinatal PTSD Questionnaire [PPQ; (45)], Posttraumatic Diagnostic Scale [PDS; (46)], Impact of Event Scale-Revised IES-R [IES-R; (47)], and Traumatic Event Scale [TES; (48)]. Outcome data were grouped according to the following time-points: up to but < 3 months post intervention; 3 months to < 6 months; 6 months to up to 12 months post intervention; and over 12 months post intervention. For outcomes measured at several time-points within these intervals, we reported the analyses separately.

### Study selection, data extraction, and risk of bias assessment

Initial screening of study titles, abstracts and full text articles was undertaken by two review authors (MF and JS) independently and in parallel. A third author (DS) conducted further independent screening at each stage for a 10% random sample. Data extraction from included studies was undertaken by MF and reviewed and checked by JS and EN. Two authors (MF and JS) used risks of bias assessment tools specific to study designs to assess quality of included studies (49, 50), independently. At each stage, all authors reviewed and resolved uncertainties through seeking additional data or clarification from the original study authors when possible, and/or review team discussion and consensus.

### Analysis strategy

The analysis began with an overview of study characteristics followed by tabulation of extracted data, in MS Excel. Overall data were synthesized narratively. Whenever there were sufficient data extracted from the included RCTs, we conducted meta-analyses using the software package, RevMan (49). We used a fixed-effect model when there were < 5 studies included in the analysis and random effects model when there were five or more studies (25). In addition to conducting overall analyses comparing TFPT (all modalities grouped together) with all comparators pooled together, we conducted separate comparisons on specific intervention modality. We also conducted a subgroup analysis based on risk of experiencing postpartum trauma: known high-risk groups (e.g., women who had a stillbirth or obstetric complications) vs. those who had had no complications. Where missing discrete outcome data may be a concern to the robustness of the result, we performed sensitivity analysis to assess the impact of potentially informative missing data (51, 52). In this analysis, weights inversely proportional to the widths of uncertainty intervals constructed under scenarios favoring each randomization group are assigned to studies with missing discrete outcome data.

As outcomes were measured with different validated scales, standardized mean difference and 95% CI were calculated for continuous outcomes. While we considered both changed and follow-up scores for analysis dependent on data availability, effect measures based on changed scores are typically more efficient (with smaller standard error) when there is no major baseline imbalance and when baseline and follow-up scores are correlated (53). While some considered SMDs of 0.2, 0.5, and 0.8 as small, medium, and large effects, the magnitude of these effects do not generally bear any relationship with their clinical importance. We calculated risk ratio (RR) and 95% CI for dichotomous data (49, 54). Statistical heterogeneity was quantified using the I-squared (*I*^2^) statistic, in addition to visual inspection of the forest plots (55). *I*^2^-values around 30% or above were interpreted as evidence of substantial levels of heterogeneity. When heterogeneity was identified, reasons for the inconsistency were explored using pre-specified subgroup analysis (based on study settings, and format of intervention delivery) if sufficient data were available.

The Grading of Recommendations Assessment, Development and Evaluation (GRADE) approach (49, 56) was used to assess the overall quality of evidence for each analysis. One of four levels—high, moderate, low, or very low—were assigned to the overall quality of evidence for each outcome, according to factors including within-study risk of bias (methodological quality), directness of evidence, heterogeneity, precision of effect estimates and risk of publication bias (49, 56). When sufficient studies were available (*n* = 10 or more), we would have constructed funnel plots to assess publication bias.

## Results

**Figure 1:**
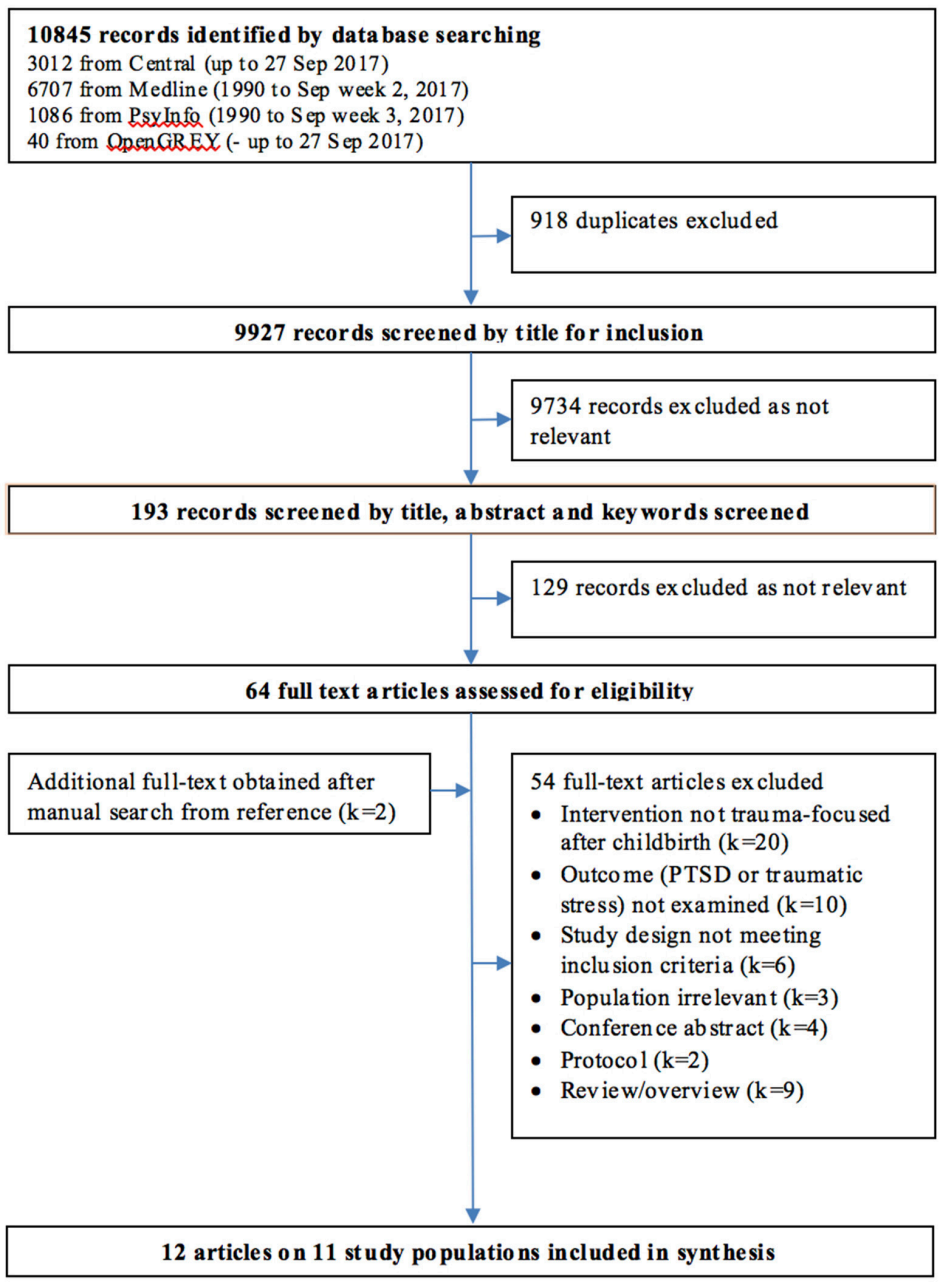
PRISMA flowchart of paper screening process.

The database search resulted in 10,845 records, of these 918 were duplicates and removed. We screened 9,927 titles and then 193 abstracts, to select 64 full-text papers for inspection. We identified two additional papers from examining the full text and reference lists of included studies. Of the total 66 papers examined, we excluded 54 due to the following reasons: irrelevant population (*k*^1^ = 3); irrelevant intervention (*k* = 20); papers reporting no usable data or empirical studies (*k* = 15); irrelevant designs (*k* = 6); and papers not reporting data on the review primary outcomes (*k* = 10). Twelve papers reporting eleven studies met all inclusion criteria and were included in this review (see Figure 1 for the PRIMA flowchart and Table 2 for a summary of included studies).

**Table 2:** Characteristics of included studies.

**Authors**	**Country**	**Setting**	**Study design**	**Recruitment dates**	**participants Inclusion (I)/exclusion (E)**	**Intervention exposure**	**Comparators**	N^*^	**Outcomes and measures**
(57)	Italy	Unclear	RCT	Unclear	I: women with a stable affective relationship E: women with pregnancy-related problems and/or diagnosed psychopathology	Expressive writing plus usual care	Usual postnatal care	64	PTSD symptoms - measured with PPQ
(58)	Italy	Hospital	RCT	2003–2007	I: women with absence of complications connected to labor and established the good health of the mother and child E: women with psychological and psychiatric pathologies	Expressive writing plus usual care	Usual postnatal care	242	PTSD symptoms - measured with PPQ
(59)	Italy	Hospital	RCT	Unclear	I: women with adequate knowledge of the Italian language; no psychiatric diagnosis in anamnesis; age ≥18, a healthy baby with the Apgar score > 7 at 1 and at 5 min. after birth.	Expressive writing about the deep emotion connected with delivery and childbirth	Usual postnatal care and neutral writing to describe daily events	113	PTSD symptoms - measured with PPQ
(60)	Switzer-land	Community	RCT	2012–2014	I: women with very preterm infant (< 32 weeks of gestation or < 1500 g birth weight) alive at the time of recruitment and group allocation (3 months), E: not speak French sufficiently to complete the questionnaires; attending regular counseling or psychotherapy sessions at the time of recruitment or group allocation (3 months).	Expressive writing with specific reference to the experience of prematurity plus usual care	Usual postnatal care	94	PTSD symptoms–measured with PPQ
(61)	Switzer-land	Hospital	RCT	2015	I: women who had EmCS with a live baby at term in the previous 6 h in the maternity department; and age ≥18; E: women with planned CS; insufficient French language; and baby transferred to neonatal intensive care unit.	Engaging in a cognitive task, the computer game Tetris, for 15 min. plus usual care	Usual postnatal care	56	PTSD symptoms–measured with PDS
(62)	Iran	Local health center	RCT	2016	I: women with perinatal loss occurred at more than 22 weeks gestational age; no history of stillbirth or miscarriage in previous pregnancies; no history of mental disorders; absence of other stressful events in the past year; age ≥18; and literacy E: failure to participate in more than one intervention session and the possible incidence of crisis or loss of relatives during the study.	4 psychological counseling sessions with the predetermined structure and content, in small groups plus usual care	Usual postnatal care	100	PTSD symptoms - measured with PPQ
(63)	Sweden	Community	RCT	2014	I: women having self-reported PTSD symptoms related to a traumatic childbirth and a TES sum score ≥30; aged ≥ 18; having access to a computer and the internet; being able to read and write Swedish; not being pregnant; not having problems requiring more urgent care; not currently participating in psychotherapy; not currently having a serious problem that would be better treated with psychiatric care; having medication, having taken the same dose for at least 1 month, with no intention to change the dose during the course of the programme; and minimum 3 months since the traumatic delivery.	Trauma-focused guided Internet-based cognitive behavior therapy plus usual care	Usual postnatal care and waiting list control	56	PTSD symptoms - measured with TES and IES-R
(64)	Austra-lia	Hospital	RCT	1996–1997	I: Women delivered at ≥35 weeks' gestation E: insufficient English to complete questionnaires, being under psychological care at the time of delivery; age < 18; or the infant needing neonatal intensive care	Stress debriefing plus usual care	Usual postnatal care	1,745	PTSD symptoms - measured with IES-R
(65, 66)	US	Hospital	RCT	2011–2012	I: English- and Spanish speaking mothers aged ≥18 years; whose infants born in 25 to 34 weeks' gestational age with >600 g or transferred to a NICUs within the first week of delivery	A manual-driven, CBT-based, trauma focused programme of 6–9 sessions plus mother-redefinition/education intervention plus usual care	Usual postnatal care and attention-control (a 45-min information session on NICU with education about parenting the premature infant)	105	PTSD symptoms - measured with DTS and MINI
(67)	Sweden	Outpatient clinic	Case studies	2001–2003	I: women with severe fear of childbirth after a previous traumatic child-birth	EMDR plus usual care	Self-comparison (before receiving EMDR)	4	PTSD symptoms - measured with TES
(68)	Canada	Hospital	RCT	Unclear	I: women who had a singleton infant born < 1500g, able to speak and read either English or French; and living within a 90-minute radius of the hospital. E: women whose infant shared the same room with another infant whose mother had already joined the study; had multiple births; infant in a highly unstable medical condition and/or likely to be transferred or discharged in < 4 weeks	6 sessions to teach mothers to recognize own and infant's signs of anxiety/ distress, and to utilize various strategies to reduce distress and respond sensitively plus usual care	Usual postnatal care and general information about infant care	98	PTSD symptoms - measured with PPQ

* *Number of participants who consented to participate in the study*.

*EmCS, Emergency Cesarean section; CS, Cesarean section; DSM-IV, Diagnostic and statistical manual of mental disorders, 4th edition; DTS, Davidson Trauma Scale (69); ICBT, Internet-based cognitive behavior therapy; IES-R, Impact of Event Scale - Revised (47); MINI, Mini-International Neuropsychiatric Interview; NICU, neonatal intensive care unit; PDS, Posttraumatic Diagnostic Scale (70); PPQ, perinatal PTSD questionnaire (45); PTSD, posttraumatic stress disorder; RCT, randomized control trial; TES, Traumatic Event Scale (48)*.

The 11 studies included 2,677 postnatal women, altogether. These studies were undertaken in the following countries: Australia (*k* = 1); Canada (*k* = 1); Iran (*k* = 1); Italy (*k* = 3); Sweden (*k* = 2); Switzerland (*k* = 2); and U.S. (*k* = 1). The U.S study reported outcome data on trauma, depression, and anxiety symptoms in postpartum women in two papers; one on post-intervention (i.e., 4–5 weeks postpartum, (65), and the other on 6-month follow up (66). All studies were RCTs, apart from one case series study (67). The earliest study was published in 2002 (57), and six out of 12 papers were published in the recent 5 years (60–63, 65, 66).

### Overview of interventions, settings, and participants

Altogether, the 11 studies tested nine interventions. These are listed below and categorized into the four modalities:

1. Exposure therapy—four studies examined two forms of expressive writing therapy, one by Horsch et al. (60) which asked women to write for 15 min per day for three consecutive days at 3 months postpartum, and another by (57, 58, 59) which instructed women to write for 15–20 min two times a day, at 3 days after giving birth.
2. TFCBT—two studies investigated a cognitive behavioral program respectively; one was delivered through the internet (63), another was an individual manual-based trauma-focused CBT program delivered by trained clinical psychology graduate students or social workers (65, 66).
3. EMDR—one case series reporting using EMDR with women who had severe fear of childbirth due to earlier trauma birth experience (67); and
4. Any other trauma-focused psychological therapies which did not fit the above categories—four interventions met this definition. These included a brief cognitive task using the computer game “Tetris” for women who had had an EmCS (61), a one-off stress debriefing session intervention for those with an uncomplicated birth (64), a group traumatic-grief counseling program for women who suffered a stillbirth experience (62), and a six-session “Cues” intervention which aimed to teach new mothers to recognize and cope better with distress in themselves and their infant (68).

The trauma-focused interventions (in addition to usual care) varied in terms of time and formats of delivery, duration, and intensity. The earliest intervention was the “Tetris” intervention, which was delivered to women within 6 h after an EmCS (61). A few other interventions, such as the expressive writing and debriefing (57–59, 64) were delivered between 1–3 days after the women had given birth. One CBT intervention was given to women 1–2 weeks after they have given birth to a preterm infant (65, 66). Two interventions were delivered to the women at 4 weeks postpartum; one study after their infants were discharged from the NICU (68), and in another after delivering a stillborn (62). A further expressive writing intervention was received by the women at 3 months postpartum (60). Two studies did not specify time since the traumatic birth (63, 67). The “dosage' and intensity of the interventions ranged from a 15-min debriefing session (64) or a 15-min Tetris session (61), to three 15-min writing times during three consecutive days (60), to 8 weekly online TFCBT sessions (63) or a nine-session TFCBT program (65, 66), lasting about 10 h in total. The length of the EMDR program in which women received individual sessions at an outpatient psychotherapy clinic was not reported (67). Most of the interventions were delivered in the hospital setting, after the women had given birth [e.g., (57–59, 61, 64)]. A couple of interventions used a combination of within-hospital and home visit delivery [e.g., (65, 66, 68)]. A further four interventions were delivered in the community; via internet (63), or by post (60), or at a local health center (62) or outpatient psychotherapy clinic (67).

Of the 10 RCTs, the comparison groups were often usual care. One study used “neutral writing” as an attention-control to compare with expressive writing (59); another study used a 45-min education session about parenting for premature infant as an active control to compare with a CBT program (65, 66).

The women targeted also varied from those having a normal or uncomplicated delivery (low risk group) (*k* = 4) (57–59, 64) to those who had a complicated or traumatic birth experience (high risk group) (*k* = 7). Some of the latter studies did not specify the trauma *per se* but targeted women who scored above a certain threshold on a self-reported PTSD symptom scale (63, 67); others focused on certain high-risk populations, including those who had an EmCS (61), a preterm birth (60, 65, 66, 68), or a stillbirth experience (62).

### Quality of included studies

We used the Cochrane Collaboration risk of bias tool for RCTs (49) to assess quality of the 10 RCTs. For the remaining case series study (67), we used a quality assessment tool specifically designed for case series studies (50). The case series was a pilot study testing the feasibility of EMDR in treating childbirth-related PTSD in four women. The small sample was recruited from a single clinic in Sweden from 2001 to 2003. Due to recruitment problems, the eligibility criteria were altered leading to the varying characteristics and symptom profiles of the participants, for example one woman was pregnant and the other three were postnatal whilst receiving treatment. While the EMDR intervention was clearly described, the specific program of treatment each woman received was less clear (such as number and frequency of sessions, duration of treatment). Only one quantitative outcome measure for PTSD symptoms (i.e., TES) was reported, supplemented by a qualitative interview, to collect follow-up outcome at 1–3 years after the last treatment session. Given the small sample size and dataset, it seemed appropriate that only non-inferential statistics were used to analyze the outcomes. However, given the time gap in the pre- and post-treatment outcome measures and the lack of information on other treatments and any other psychosocial factors which might have affected the women's prognosis, the conclusion drawn on the feasibility of EMDR treatment was found to be less than substantiated.

Our overall evaluation of the risk of bias of the RCTs is presented in Table 3. Sequence generation was adequately described in five studies, unclear in four, and regarded as high risk in one Iranian study (62) given the somewhat contradictory accounts of the randomization process. Two studies, both from Switzerland (60, 61), were rated as low risk in terms of allocation concealment, seven as unclear, one as high risk with baseline assessment conducted only after allocation (62). Masking of participants and trial therapists was not possible in nine trials and we rated these as moderate risk in blinding. One remaining trial used an attention-control comparison group to mask group allocation to participants and used blinded outcome assessors; it was rated as low risk (68). Regarding incomplete outcome data due to attrition or missing data, two studies (60, 61) were at low risk of bias because ITT analyses were conducted with multiple imputation models or nearly complete follow-up data was reported. Six RCTs were rated as unclear because the method used for handling of missing data was unclear (some suggested to have performed ITT analysis, when results from seemingly “complete case analysis” were reported) and two as high with no details of missing outcome data. Only three RCTs had published a protocol or trial registration prior to commencing recruitment. These, plus one further study which reported all outcome data (60) were regarded as low risk in selective outcome reporting; the remaining six were rated as unclear. Overall, studies largely reported outcomes using validated scales; many were self-reported measures. Although most of the trials were published fairly recently, not all of them were reported following the CONSORT guidelines, including a CONSORT flowchart showing the participant pathway through the trials. Only six studies reported obtaining research ethics approval. Information on sources of funding and/or conflict of interests was scarcely reported. Only four studies (62, 64–66, 68) reported training and qualification requirement of the clinicians/therapists and some of these also reported considerations of treatment fidelity checking. We considered that these factors contributed to other sources of bias and subsequently rated four studies as unclear risk and the remaining six as low risk.

**Table 3:** Quality assessment of included RCTs.

**Study**	**Study design**	**Sequence generation**	**Allocation concealment**	**Blinding of participants, personnel & assessors**	**Incomplete outcome data**	**Selective reporting**	**Any other sources of bias**
(57)	RCT	?	?	?	×	?	?
(58)	RCT	?	?	?	×	?	?
(59)	RCT	✓	?	?	?	?	?
(60)	RCT	✓	✓	?	✓	✓	✓
(61)	RCT	✓	✓	?	✓	✓	✓
(62)	RCT	×	×	?	?	?	?
(63)	RCT	✓	?	?	?	?	✓
(64)	RCT	?	?	?	?	?	✓
(65)^*^)	RCT	✓	?	?	?	✓	✓
(68)	RCT	?	?	✓	?	✓	✓

* *Risk of bias assessment was conducted on the main paper*.

*✓Low risk of bias; × High risk of bias; ? Unclear risk of bias*.

### Intervention effectiveness on PTSD and posttraumatic stress symptoms

#### Presence of posttraumatic stress disorder diagnosis or clinically significant levels of posttraumatic stress symptoms

##### Short term: up to 3 months postpartum

**Figure 2:**
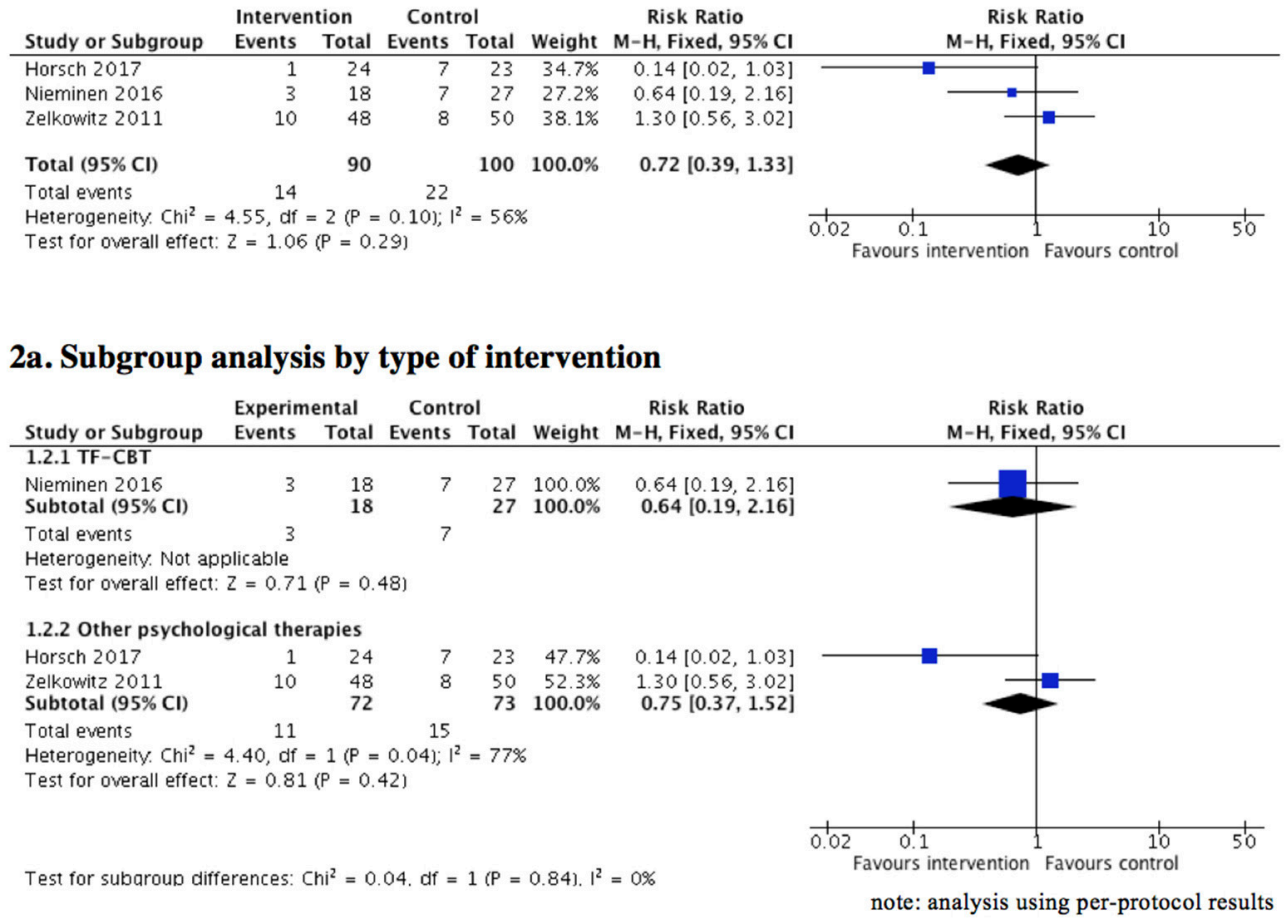
Effect of psychological therapies on clinically significant level of posttraumatic stress symptoms (short term: up to 3 months postpartum).

Three studies (61, 63, 68), including postpartum women who had experienced a traumatic birth or obstetric/neonatal complications, investigated the effectiveness of TFPT on reducing PTSD symptoms to the extent of below cut-off scores and/or clinically significant levels of PTSD symptoms (as defined by the study authors). A meta-analysis (Figure 2) using “per-protocol” results showed no significant difference between the intervention group and usual care/inactive comparison group (3 RCTs, *n* = 190, RR = 0.72, 95% CI = 0.39 to 1.33, fixed effect, GRADE quality of evidence = very low). The sensitivity analysis comparing fixed and random-effects estimates showed similar results. We noted that outcome data were missing for 41 of the total of 233 women randomized in the three studies. One trial (61) reported both ITT and per-protocol results, while the other two reported results from “complete case analysis” (i.e., including only those individuals with outcome data observed at follow-up). To assess the potential impact of the missing data on the result, a sensitivity analysis was performed that found a fractionally stronger but non-statistically significant effect in favor of the intervention group (RR = 0.68, 95% CI = 0.24–1.95). Hence, the same conclusion holds, irrespective of the presence of the potentially informative missing data.

Of note, clinical heterogeneity of these studies was high considering the different intervention approaches: one used a computerized cognitive task (61); one a TFCBT (63); and the remainder a combined training intervention (68). A subgroup analysis (Figure 2) was performed to further assess the differences in the proportion of women with clinically significant levels of posttraumatic stress symptoms between different types of TFPT compared to inactive controls. While heterogeneity of the results remained (I-squares = 77%) between the trials involving Tetris and combined training (61, 68), the results showed no significant difference in the outcomes between the intervention group and control group (2 RCTs, *n* = 145, RR = 0.75, 95% CI = 0.37–1.52, fixed effect, GRADE quality of evidence = very low). The sensitivity analysis comparing fixed and random-effects estimates showed similar results. Non-significant results were also found in the corresponding sensitivity analysis, considering the impact of missing data (RR = 0.70, 95% CI = 0.19–2.58).

##### Medium term: 3–6 months postpartum

One RCT (60) involving 61 women who gave birth to very preterm infants (< 32 weeks of gestation or < 1500 g birth weight) reported no significant difference in the risk of clinically significant levels of PTSD symptoms (as measured with PPQ > 6) at 4 months postpartum between women who had received the expressive writing intervention and those who had usual postnatal care.

##### Long term: 6 months or longer postpartum

The same RCT (60) reported no significant group difference regarding clinically significant levels of posttraumatic stress symptoms at 6 months postpartum between the intervention and usual care group. One further study (64) examined posttraumatic stress symptoms at the three follow-up time points within one year postpartum (at 2, 6, or 12 months after giving birth) and showed no significant difference between the debriefing intervention group and the control group. However, the results of each assessment time were not presented separately; thus, it was impossible to include the study data in the current analysis.

#### Severity of posttraumatic stress symptoms (continuous scores)

##### Short term: endpoint scores in PTSD symptoms up to 3 months postpartum

**Figure 3:**
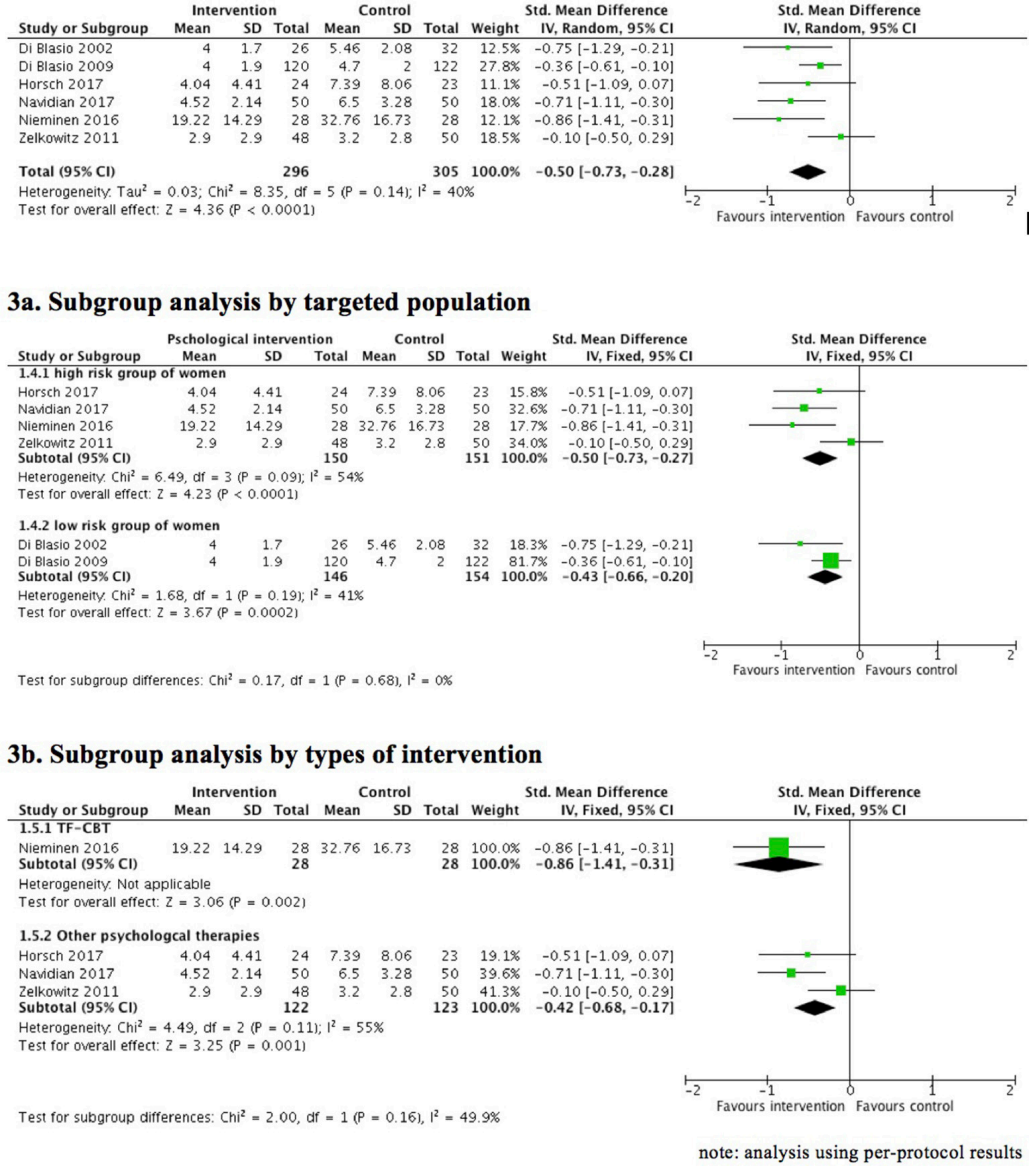
Effect of psychological therapies on posttraumatic stress symptoms scores (short term: up to 3 months postpartum).

Six trials provided data for this analysis. A meta-analysis using per-protocol analysis data (Figure 3) shows that posttraumatic stress symptoms in the early postnatal period were lower overall in the intervention group compared to the control group; the differences were statistically significant (6 RCTs, *n* = 601, SMD = −0.50, 95% CI = −0.73 to −0.28, random effect, GRADE quality of evidence = moderate). The results were similar between fixed and random-effects estimates. Intention-to-treat meta-analysis was not possible because only one trial (61) provided ITT results using multiple imputation to handle missing outcome data.

These results were based on trials with potential clinical heterogeneity in terms of different intervention modalities and targeted populations. A subgroup analysis by targeted population (Figure 3) showed significantly lower posttraumatic stress symptoms in the early postnatal period in the intervention group compared to the control group both in the high risk group (4 RCTs, *n* = 301, SMD = −0.50, 95% CI = −0.73 to −0.27, fixed effect [similar results obtained with random-effect estimate], GRADE quality of evidence = moderate) and the low risk group (i.e., women who had healthy pregnancies and uncomplicated birth). Although the point estimate of the effect was consistently in favor of psychological interventions, there was considerable statistical heterogeneity between trials with women at high risk of traumatic birth (*I*^2^ = 54%). In addition, two RCTs examined PTSD symptoms in women of low risk group (57, 58), and the results favored expressive writing in addition to usual care compared to usual care alone (2 RCTs, *n* = 300, SMD = −0.43; 95% CI = −0.66 to −0.20, fixed-effect [similar results obtained with random-effect estimate], GRADE quality of evidence = moderate).

A subgroup analysis was further performed to assess the effectiveness of various intervention modalities on PTSD symptoms among women at high risk of traumatic birth. One study investigated internet-based TFCBT targeting women with self-reported posttraumatic stress symptoms related to childbirth (63), which showed that PTSD symptoms were significantly lower in the intervention group compared to the usual postnatal care. The remaining three studies investigated interventions not fitting the modalities of exposure, CBT, or EMDR (60, 61, 68). Analyses of subgroup data provided some evidence that these interventions yielded greater improvement in PTSD symptoms, compared with inactive controls. Of note, the heterogeneity of the trial results remained (I-squares = 60%) between the trials (3 RCTs, *n* = 245, SMD = −0.42, 95% CI = −0.68 to −0.17, fixed effect [similar results obtained with random-effect estimate], GRADE quality of evidence = low) (see Figure 3).

##### Short term: mean score changes in posttraumatic stress symptoms from baseline to the follow-up time point (up to 3 months postpartum)

**Figure 4:**
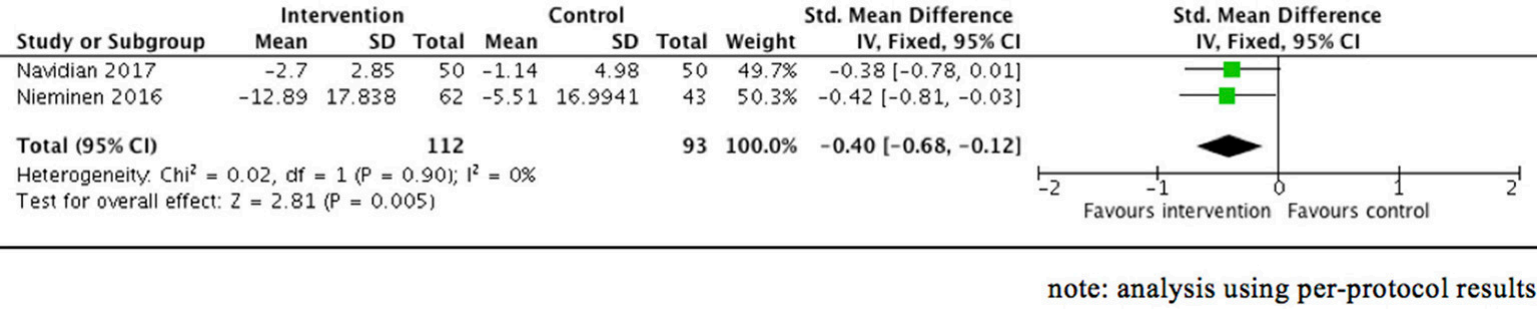
Effect of psychological therapies on posttraumatic stress symptom—mean score changes from baseline (short term: up to 3 months postpartum).

Four RCTs (62, 63, 65, 66, 68) involving women who had traumatic childbirth and/or neonatal complications presented the mean score changes in PTSD symptoms from baseline to the follow-up point in the early postnatal period. Meta-analysis (Figure 4) was only possible with usable per-protocol data obtained from two of the four RCTs (62, 65), which showed a statistically significant reduction of posttraumatic stress symptom score in the intervention group compared to the control group (2 RCTs, *n* = 205, SMD = −0.40, 95% CI = −0.68 to −0.12, fixed effect [similar results obtained with random-effect estimate], GRADE quality of evidence = low). Another trial (63) also showed greater reduction of PTSD symptoms but neither standard deviation, 95% confidence interval nor *p*-value were presented. There was, however, one trial (68) involving 98 women, which showed no difference in mean score changes regarding posttraumatic stress symptoms at 6–8 weeks between the intervention group and the control group.

##### Medium term: endpoint scores in posttraumatic stress symptoms at 3–6 months postpartum

**Figure 5:**
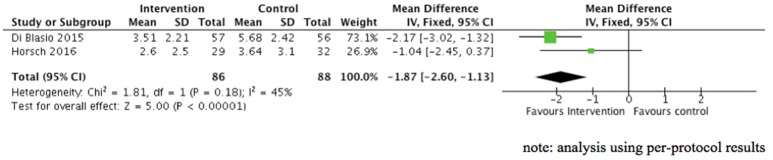
Effect of psychological therapies on posttraumatic stress symptom scores (medium term: 3–6 months postpartum).

Two studies, both investigating expressive writing in postpartum women, provided data for this analysis (59, 60). Figure 5 shows that posttraumatic stress symptoms at 3–6 months postpartum overall were significantly lower in the expressive intervention group compared to the control group (2 RCTs, *n* = 174, SMD = −1.87, 95% CI = −2.60 to −1.13, fixed effect [similar results obtained with random-effect estimate], GRADE quality of evidence = low). These results were based on trials with potential clinical heterogeneity in terms of different targeted population, i.e., women who gave birth to very preterm babies (60) and women who delivered a healthy baby following an uncomplicated birth (59).

##### Medium term: mean score changes in posttraumatic stress symptoms from baseline to the follow-up time point (3–6 months postpartum)

Two RCTs examined mean score changes in posttraumatic stress symptoms from baseline to 3–6 months postpartum (60, 59), of which only one RCT (59) presented data for statistical analysis. The results indicated that there was significantly greater decrease in PTSD symptoms from baseline to 3 months postpartum in women who had expressive writing compared to women who had neutral writing. One RCT (60) also showed that the decrease in PTSD symptoms from baseline to 4 months postpartum was larger in the expressive writing group compared to the usual postnatal care group, although data (standard deviation or 95% confidence interval) were not presented to assess the statistical significance.

##### Long term: endpoint scores in posttraumatic stress symptoms at 6 months or longer postpartum

Only one RCT involving 54 women who gave birth to a preterm baby, provided 6-month outcome data here (60). There was no significant difference in PTSD symptoms at 6 months postpartum between women across the intervention and the control groups.

##### Long term: mean score changes in posttraumatic stress symptoms from the baseline to the follow-up time point (6 months postpartum or more)

One RCT (66) involving 105 women in the US whose babies were born preterm or transferred to a NICU indicated that the average difference in the mean change of PTSD symptoms scores at 6 months postpartum in the TFCBT group was larger compared to the control group (*p* < 0.001). One RCT (60) also showed that decrease in PTSD symptoms from baseline to 6 months postpartum was larger in the expressive writing group compared to the control group, although data (standard deviation or 95% confidence interval) were not presented to assess the statistical significance.

## Discussion

The aim of this review was to quantitatively synthesize data obtained from clinical trials testing the effectiveness of TFPT to ameliorate women's PTSD symptoms following childbirth. Eleven studies, providing data on 2,677 women, were included in the review. All studies were published in the last 15 years, with two-thirds published during the last decade, thus reflecting the growing recognition and interest in the subject. The studies included mostly focused on delivering TFPT to postnatal women shortly after they had given birth (whether complicated or not). A range of TFPT was investigated, some of which were already commonly used in other clinical populations, such as veterans from the armed forces and victims of crimes. These included exposure therapies using expressive writing, EMDR, and TFCBT (26–28). Others were relatively novel, such as using the computerized cognitive task “Tetris” (61) and internet-based TFCBT (63). A range of validated and widely-used self-reported outcome measures in PTSD symptoms was used across the studies.

Overall, our analyses indicated that TFPT (regardless of intervention modalities) are effective in reducing PTSD symptoms in the early postnatal period, both in short term (up to 3 months postpartum) and medium term (3–6 months postpartum). Subgroup analyses further showed that the computerized cognitive task “Tetris” (61), counseling (62), combined training (68), and TFCBT (63) were particularly effective for women at high risk of experiencing a traumatic birth; while expressive writing had beneficial effects in reducing PTSD symptoms in those with an uncomplicated birth. Expressive writing was also found to be effective in reducing PTSD symptoms in postpartum women in the medium term (i.e., 3–6 months), regardless of whether they had had an objectively traumatic birth (high risk group) or not (low risk group) (59, 60). However, the clinical significance is not yet clear because there is no robust evidence to suggest whether TFPT can also improve women's recovery from clinically significant PTSD symptoms. There were limited usable medium- and long-term follow-up data, precluding meta-analysis on PTSD symptoms beyond 6-month follow up.

This systematic review and meta-analysis analyzed PTSD symptoms and diagnosis separately and generally found more favorable results of the TFPT for PTSD symptoms but not diagnosis. It is worth-noting that dichotomous data did not necessarily represent clinical caseness as most studies did not report using well-established cut off points. Another plausible reason behind the difference may stem from the symptom profile of the included samples, which comprised a good proportion of women who had an uncomplicated birth (or no exposure to objective birth trauma). This baseline profile might have rendered the prevalence of women meeting the caseness threshold too low to be detected in the study sample.

While most of the TFPT targeting postpartum women examined in the review are based on well-established models and have been widely applied to other clinical populations [e.g., (32, 36)], it is worth-noting that all reported interventions had been adapted or specifically designed to suit the needs of postpartum women. These modifications included: scheduling the intervention sessions/implementation time to fit with maternity care demands [e.g., (61, 64)]; intervention content designed to meet the care needs of the newborns [e.g., (68)]; and intervention delivered *in situ* at the maternity units or the women's home [e.g., (60, 63, 68)]. Furthermore, as the women usually received specific TFPT on top of usual postnatal care, this highlights the importance of the maternity/physical care input in parallel to addressing the complex clinical needs, such as adequate pain control or help with the initiation of breastfeeding.

While the results of the meta-analyses (combining three different modalities of TFPT together, i.e., exposure therapy, TFCBT, and other modalities) support the use of TFPT, it remains unclear if any particular type of TFPT is superior for any specific subgroup of women with PTSD symptoms following childbirth (low risk vs. high risk groups). We therefore recommend that the results of the meta-analyses are considered together with the women's specific circumstances and the clinical settings when determining the treatment of choice.

### Strengths, limitations, and recommendations

To the best of our knowledge, this is the first systematic review on TFPT for postpartum women. We were able to synthesize data from 11 studies, including several published in the last 2 years. While the wide range of interventions, undertaken across the Western and Middle East cultures may have contributed to heterogeneity in the planned analyses, this may also have enhanced generalizability of the review findings. The lower numbers of studies available for the pre-specified outcomes limited our model choice (to fixed-effect model as per our published systematic review protocol) (25), and, in turn, the generalizability of our review findings. This body of research seems to reflect the growing understanding of PTSD following childbirth and essentially, the development and provision of TFPT that is adapted for the postnatal context.

We acknowledge several limitations of this review. First, the available data underpinning our primary outcome were limited. Similarly, follow-up data were sparse, limiting analyses on outcomes beyond 6-month follow-up. Second, our efforts to categorize the interventions into four modalities (i.e., exposure therapy, TFCBT, EMDR, and other TFPT) and the targeted populations into either high risk or low risk groups, often resulted in only one or two trials reporting on any particular intervention type or population in subgroup analyses. Third, despite our intention and effort to search for studies which might have included postpartum women as participants with other clinical populations, no such studies were identified. It is possible that data from postpartum women were not reported separately. Fourth, there were no details from included studies to indicate if some of the women had prior untreated or treatment-resistant PTSD, and any other comorbidity issues commonly seen in other PTSD clinical population, such as substance misuse. Although the history of trauma(s) might not be accurately measured because it was in most, if not all, studies based on self-report, something that is very common in this research domain. However, given that the studies in our review were RCTs (except for one case series), the probability of including participants with such a history should have been equally high for both intervention and control groups, if the randomization was successful. Fifth, although we intended to conduct random-effects meta-analyses using mean score changes primarily (as this approach would have taken baseline measurement into consideration, which is particularly important in small trials) (49, 54), usable data were limited. We therefore presented fixed-effect meta-analyses using both mean score changes and endpoint scores which limits the generalizability of our findings to the included studies (71). However, sensitivity analyses comparing the fixed- and random-effects estimates of the intervention showed similar results, indicating any small-study effects have little effect on the intervention effect estimate. Sixth and lastly, it is worth noting that the quality of some of the included studies, in particular their reporting of randomization sequence generation, allocation concealment, blinding, and other sources of bias such as ethics and funding arrangement, were considered to be unclear or as having a high risk of bias. The quality of the evidence of some of the results should therefore be interpreted in light of the risk of bias assessment of the data source.

A common limitation of the studies reported here is the fact that normative experiences during the postpartum period, such as fatigue or increased vigilance in relation to the baby (72) can interfere with the correct measurement of postpartum PTSD. For example, two or more hyperarousal symptoms were reported by half of the women who did not experience traumatic births, thus highlighting the poor specificity of hyperarousal symptoms (73). It is therefore important to examine to what extent measures used in other populations suffering from PTSD are valid in women after childbirth and to develop measures that specifically target this population.

Future research should include larger trials with longer follow-ups that compare different treatment modalities, careful consideration of the optimal intervention design (such as timing of and modifications for implementation), assessment of acceptability and satisfaction with interventions, and investigation into population and intervention factors that mediate differential treatment effectiveness. Further research should also focus on establishing how best to provide healthcare professionals with training in order to facilitate implementation of TFPT for women, and the economic costs of training and implementation.

## Conclusion

Results of this systematic review suggest that TFPT are effective in reducing PTSD symptoms in the early postnatal period in the short and medium term. However, there is no robust evidence to suggest whether TFPT can also improve women's recovery from clinically significant PTSD symptoms. Further larger studies, distinguishing between high and low risk groups, with longer-term follow-up to explore different modalities of trauma-focused interventions to suit women with postnatal PTSD symptoms are necessary.

## author contributions

MF proposing and designing the review, protocol development, paper screening, data extraction, quality appraisal of papers, conducting statistical analysis and interpretation of data, and writing the review. AH revising review protocol, interpretation of data, writing and reviewing the review. EN design of the review and protocol development, checking accuracy of data extraction and conducting statistical analysis and reviewing the review. DB design of the review and protocol development, writing and reviewing the review. DS design of the review and protocol development, paper screening, writing and reviewing the review. JS proposing and designing the review, protocol development, paper screening, checking accuracy of data extraction, quality appraisal of papers, analysis and interpretation of data, writing and reviewing the review.

### Conflict of interest statement

The authors declare that the research was conducted in the absence of any commercial or financial relationships that could be construed as a potential conflict of interest.

## Footnotes

^1^*k* = number of studies.
